# WNT9A Is a Conserved Regulator of Hematopoietic Stem and Progenitor Cell Development

**DOI:** 10.3390/genes9020066

**Published:** 2018-01-29

**Authors:** Jenna Richter, Edouard G. Stanley, Elizabeth S. Ng, Andrew G. Elefanty, David Traver, Karl Willert

**Affiliations:** 1 Department of Cellular and Molecular Medicine, University of California, San Diego, CA 92037, USA; jrichter@ucsd.edu; 2 Murdoch Childrens Research Institute, The Royal Children’s Hospital, Parkville, VIC 3052, Australia; ed.stanley@mcri.edu.au (E.G.S.); elizabeth.ng@mcri.edu.au (E.S.N.); andrew.elefanty@mcri.edu.au (A.G.E.); 3 Department of Anatomy and Developmental Biology, Faculty of Medicine, Nursing and Health Sciences, Monash University, Clayton, VIC 3800, Australia; 4 Department of Paediatrics, Faculty of Medicine, Dentistry and Health Sciences, University of Melbourne, Parkville, VIC 3052, Australia

**Keywords:** hematopoiesis, hematopoietic stem cells, Wnt signaling, WNT9A, human embryonic stem cells, induced pluripotent stem cells

## Abstract

Hematopoietic stem cells (HSCs) differentiate into all cell types of the blood and can be used therapeutically to treat hematopoietic cancers and disorders. Despite decades of research, it is not yet possible to derive therapy-grade HSCs from pluripotent precursors. Analysis of HSC development in model organisms has identified some of the molecular cues that are necessary to instruct hematopoiesis in vivo, including Wnt9A, which is required during an early time window in zebrafish development. Although bona fide HSCs cannot be derived in vitro, it is possible to model human hematopoietic progenitor development by differentiating human pluripotent stem cells to hematopoietic cells. Herein, we modulate WNT9A expression during the in vitro differentiation of human embryonic stem cells to hematopoietic progenitor cells and demonstrate that WNT9A also regulates human hematopoietic progenitor cell development in vitro. Overexpression of WNT9A only impacts differentiation to CD34^+^/CD45^+^ cells during early time windows and does so in a dose-dependent manner. The cells that receive the Wnt signal—not the cells that secrete WNT9A—differentiate most efficiently to hematopoietic progenitors; this mimics the paracrine action of Wnt9a during in vivo hematopoiesis. Taken together, these data indicate that WNT9A is a conserved regulator of zebrafish and human hematopoietic development.

## 1. Introduction

Hematopoietic stem cells (HSCs) give rise to all blood cells. This property is exploited for therapeutic use; HSC transplants from bone marrow or peripheral blood are commonly used to treat hematopoietic cancers and disorders [1,2]. Induced pluripotent stem cell technology has made it possible to derive patient-matched pluripotent cells that are theoretically capable of differentiating into HSCs [3,4,5]. These cells represent a potential source of HSCs for each patient in need of a transplant, which would be a significant advancement in the field of regenerative medicine. However, it is not yet possible to derive therapy-grade HSCs in vitro from pluripotent precursors [4,5,6]. Current protocols yield hematopoietic cells with limited repopulation capacity, subpar ability to differentiate into all blood lineages, or utilize the enforced expression of hematopoietic transcription factors to induce hematopoietic fate [6,7,8,9,10,11,12,13,14]. Thus, many researchers are turning to vertebrate model organisms such as mouse and the zebrafish to identify critical cues that instruct HSC identity in vivo, in order to determine whether these signals can be leveraged to generate hematopoietic stem and progenitor cells from pluripotent precursors in vitro.

Multiple studies have identified specific molecules required for HSC development in zebrafish. These studies have shown great translational potential for increasing the functionality and viability of human HSCs derived from umbilical cord blood [15,16,17]. Therefore, many of the signals that govern HSC development are highly conserved across vertebrate species. However, it remains to be seen if this is true of many other signaling cues that have been identified as critical regulators of hematopoietic development in model organisms. Furthermore, how these regulators impact human hematopoietic development during in vitro differentiation from pluripotent precursors remains unknown.

The molecular cues governing embryonic HSC development are highly conserved amongst vertebrates, though the anatomical sites of hematopoiesis can vary between organisms [18]. In all vertebrate organisms, HSCs develop from a specialized population of hemogenic endothelium derived from the mesodermal lineage in vivo and this is recapitulated in vitro in human cells [19,20,21,22,23,24,25,26,27]. In vivo, these cells originate as lateral populations of mesoderm that migrate to the midline underneath the somites to eventually form the vasculature, which contains the hemogenic endothelium [28,29,30,31]. The somites provide many inductive cues to migrating endothelial cells to instruct hematopoietic endothelium specification [20,32,33,34,35]. We and others have recently identified Wnt/β-catenin signaling to be one such molecular cue that is required for the development of HSCs in zebrafish [15,36].

Wnt/β-catenin signaling has been implicated in directing hematopoietic stem and progenitor cell development, both in vivo and in vitro [37,38,39,40,41]. Wnt ligands are lipid-modified secreted growth factors that bind to Frizzled (Fzd) receptors to activate intracellular signaling cascades [42]. Upon Wnt–Fzd binding, a protein complex that targets β-catenin for proteasomal degradation is dissociated, stabilizing β-catenin, and allowing its translocation to the nucleus, where it interacts with co-activators to initiate the expression of target genes. In zebrafish HSC development, Wnt/β-catenin signaling is required in a temporally restricted manner during early endothelial cell migration, after which it is dispensable [36]. We have shown that Wnt9a is the mediator of this critical Wnt signal. Wnt9a is secreted by the somites and received by adjacent hemogenic endothelial cells, which later proliferate in response to the activation of the Wnt target gene, *myca* [36]. Similar studies in differentiating mouse embryonic stem cells also identified defined time windows during which BMP4, Wnt or Activin signaling were required for mesoderm induction [43]. After this time window, Wnt signaling is dispensable for HSC development.

Hematopoietic progenitor cells can be derived from human embryonic stem cells (hESCs), which can be used as in vitro surrogates of human hematopoietic development [44,45]. Protocols to derive hematopoietic progenitors from pluripotent precursors transition cells through mesodermal, endothelial, and hematopoietic stages that are similar to the stage progressions that occur in vivo [6]. These developmental transitions are governed by molecular cues that are conserved across vertebrates [18]. A notable difference between the in vitro system and normal in vivo hematopoietic development is the absence of the highly organized spatial architecture that exists in a developing embryo. Cellular migrations and tissue movements usually occur in a tightly regulated manner in the embryo; in the in vitro model, cells do not exist in highly organized tissues, and do not move in these prescribed fashions. As a result, cells are likely receiving important molecular cues at slightly asynchronous times. Despite these differences, the in vitro differentiation of hematopoietic progenitors is an accessible platform on which to dissect signaling inputs during human hematopoiesis.

Utilizing the in vitro hematopoietic differentiation system as a model for human hematopoietic development, we show here that (1) Wnt positively regulates the differentiation of human progenitors; (2) WNT9A impacts in vitro human hematopoiesis in a time-dependent manner; (3) WNT9A has a dose-dependent effect on hematopoietic differentiation; (4) in vitro differentiation dynamics in response to WNT9A mimic the paracrine nature of the Wnt9a signal observed in zebrafish.

## 2. Materials and Methods 

### 2.1. Human Pluripotent Stem Cells Culture

All experiments described in this study were approved by a research oversight committee (IRB/ESCRO Protocol #100210, PI Willert). Human embryonic stem cell H9 (WA09, NIH Registration Number 0062) cells were obtained from WiCell (Madison, WI, USA). *H9-RUNX1c: Green fluorescent protein (GFP); SOX17:mCherry* (referred to hereafter as RUNX1c:GFP) knock-in reporter cells were previously described [46]. Cells were maintained in Essential 8 (E8) media (DMEM/F12 supplemented with l-ascorbic acid, selenium, transferrin, NaHCO_3_, insulin, Transforming Growth Factor (TGF) β1, and Fibroblast Growth Factor (FGF) 2) as described previously [47]. Transgenic cell lines were maintained in 2 μg/mL puromycin (Thermo Fisher Scientific, Waltham, MA, USA). Cells were passaged every five days with TrypLE Express (Thermo Fisher Scientific) and seeded onto Matrigel (Corning, Corning, NY, USA) coated tissue culture dishes in medium containing 1 µM ROCK inhibitor (Y-27632 dihydrochloride; Enzo Life Sciences, Farmingdale, NY, USA).

### 2.2. Generation of Transgenic Human Pluripotent Stem Cells Lines

The doxycycline-inducible WNT9A hESC (H9) cell line was generated by co-transfecting PiggyBac transposons [48] encoding the transgene with a plasmid constitutively expressing a hyperactive version of the PiggyBac transposase [49,50,51] using GeneIn transfection reagent (GlobalStem, Rockville, MD, USA) according to manufacturer directions. On the day of transfection, 10% fetal bovine serum (FBS; Peak Serum, Inc., Fort Collins, CO, USA) was added to E8 growth media. Cells were selected for transgene integration with 4 μg/mL puromycin (Thermo Fisher Scientific). The Wnt reporter cell line was generated by infecting H9 cells with a lentivirus containing a previously cloned lentivector (Addgene Plasmid #24304; Cambridge, MA, USA). Infected cells with high expression of mCherry were isolated using fluorescence-activated cell sorting on a BD FACSAria II (BD Biosciences, San Jose CA, USA), and used for differentiation within ten passages of lentiviral infection.

### 2.3. Reverse Quantitative Polymerase Chain Reaction

Synthesis of RNA and complementary DNA (cDNA) was performed using the Zymo Direct-zol (Zymo, Irvine, CA, USA) and iScript Supermix (Bio-Rad, Hercules, CA, USA) kits, and quantitative PCR (qPCR) was performed using iTaq Universal SYBR Green Supermix (Bio-Rad) according to the manufacturer’s recommendations and analyzed using the 2^−ΔΔCt^ method [52]. Sequences of primers used are shown in Table 1.

### 2.4. Hematopoietic Differentiation

Human embryonic stem cells (hESCs) were differentiated to hematopoietic progenitor cells as described by Ng et al. [44,45]. Briefly, on the day before beginning the differentiation, cells were passaged at a high density (approximately 1.5 × 10^5^ cells/cm^2^) into E8 media with 2.5 µM ROCK inhibitor (Y-27632 dihydrochloride) onto Matrigel-coated tissue culture dishes using TrypLE Express. Twenty-four hours later, cells were dissociated using TrypLE Express and resuspended in STEMdiff APEL2 medium (Stemcell Technologies, Vancouver, BC, Canada) supplemented with 40 ng/mL BMP4, 40 ng/mL SCF, 20 ng/mL VEGF (R&D Systems, Minneapolis, MN, USA), 10 ng/mL FGF2 (Peprotech, Rocky Hill, NJ, USA), and 2.5 µM ROCK inhibitor. Then, 3 × 10^3^ cells were seeded per well of a low-attachment U-bottom 96-well plate in 100 µL of media and incubated for seven days in a 37 °C/5% CO_2_ incubator. After seven days, embryoid bodies (EBs) were transferred to gelatin-coated plates and one volume of media with growth factors was added. Cells were harvested and analyzed after 14 days of differentiation (unless otherwise indicated). When indicated, CHIR98014 (CHIR) and C59 (Selleck Chemicals, Houston, TX, USA) were added to the differentiation at the indicated dosage in a final concentration of 0.1% dimethyl sulfoxide (DMSO). Doxycycline (Sigma–Aldrich, St. Louis, MO, USA) was added to induce transgene expression when indicated at the specified dosage.

### 2.5. Flow Cytometry and Fluorescence-Activated Cell Sorting 

Cells were dissociated using TrypLE Express and resuspended in flow cytometry and fluorescence-activated cell sorting (FACS) buffer. When indicated, cells were stained with anti-CD34 and anti-CD45 antibodies (BioLegend, San Diego, CA, USA). Cells were analyzed by standard means on a BD FACSCanto (BD Biosciences) or a BD LSRFortessa (BD Biosciences). Cells were sorted on a BD FACSAria II according to standard procedures. Flow cytometry data was analyzed using FlowJo software (FlowJo, Ashland, OR, USA).

### 2.6. Blue Sepharose Pull Down from Conditioned Media

Media treated for the indicated length of time after treatment with 50 ng/mL doxycycline were collected from cells, and TritonX-100 (Sigma-Aldrich) and Tris-Cl pH 7.5 were added to final concentrations of 1%, and 20 mM, respectively, and sterile filtered. Blue Sepharose bead slurry (1:1 ratio of phosphate-buffered saline (PBS): beads) was added to conditioned media in a 1:100 dilution and rotated at room temperature for 1 h. The beads were spun down at 1000 × *g* for 3 min and washed three times with 1% 3-[(3-cholamidopropyl)dimethylammonio]-1-propanesulfonate (CHAPS) in PBS. The washed beads were diluted in 1X protein loading buffer, heated at 95 °C for 5 min, then used for immunoblotting with the indicated antibodies.

### 2.7. Western Blot

Cells were lysed in 1% TritonX-100, 150 mM NaCl, and 50 mM Tris, pH 8.0 supplemented with protease inhibitor cocktail (Roche, Basel, Switzerland) on ice, and centrifuged at 20,000× *g* for 10 min at 4 °C to remove insoluble material. Twenty micrograms of cleared lysate was run on a SDS-polyacrylamide gel and transferred to nitrocellulose, blotted with antibodies against the indicated target protein, which was detected using enhanced chemiluminescence.

### 2.8. Antibodies

The anti-WNT9A antibody was generated against a fusion protein comprised of Glutathione S-Transferase (GST) fused to 70 amino acids of the zebrafish Wnt9a protein (zWnt9a, amino acid sequence LAPFHEIGKQLKQRYETSVKVASSTNEATGEGEISQSRSQSQQPPQPDIPRTPDLLHIEDSPSLERPHRD), or 65 amino acids of the human WNT9A protein (hWNT9A, amino acid sequence LAPFHEVGKHLKHKYETALKVGSTTNEAAGEAGAISPPRGRASGAGGSDPLPRTPELVHLDDSPS). These GST-WNT9A fusion proteins were generated by subcloning the open reading frame encoding the above amino acids of zWnt9a or hWNT9A into pGEX4T-1 (GE Healthcare Life Sciences, Marlborough, MA, USA), expressing the protein in BL21 bacteria induced with 1 mM IPTG, and purifying the protein using glutathione Sepharose beads (GE Healthcare Life Sciences). This purified fusion protein was injected into rabbits at Lampire Biological Laboratories (Pipersville, PA, USA) according to standard practices. Serum from rabbits immunized with the GST-zWnt9a protein was depleted of anti-GST antibodies by affinity purification over a GST column, and enriched for anti-Wnt9a antibodies by affinity purification over a GST-Wnt9a column. This enriched material was utilized for immunoblotting at a 1:1000 dilution. This Wnt9a antibody was reactive with both the zebrafish and human WNT9A (data not shown) and was utilized in the immunoblot shown in Figure 3C. Serum from rabbits immunized with the GST-hWNT9A protein was purified over a MBP-hWNT9A column and concentrated over a 30 kDa molecular weight cutoff column. This material was utilized for immunoblotting at a 1:5000 dilution in the blot shown in Figure 2B. The anti-β-actin antibody was purchased from Sigma–Aldrich (Cat. No. A2228) and utilized for immunoblotting at a 1:10,000 dilution.

## 3. Results

### 3.1. Wnt/β-Catenin Signaling Regulates in Vitro Development of Human Hematopoietic Progenitor Cells

**Figure 1:**
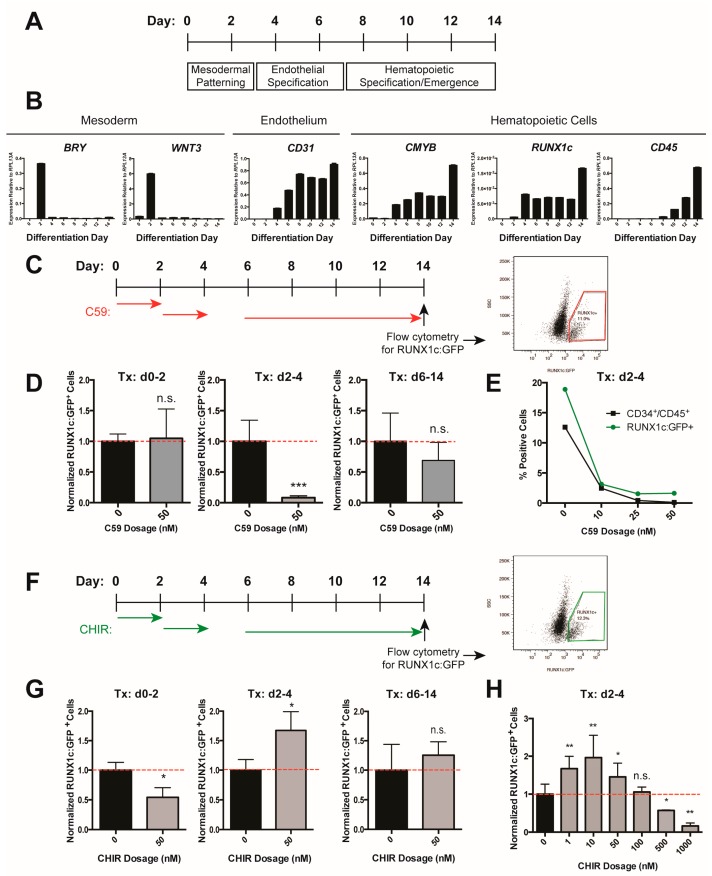
Wnt/β-catenin signaling positively regulates in vitro hematopoietic progenitor development. (**A**) Schematic of differentiation protocol; (**B**) Reverse transcription quantitative PCR (RT-quantitative PCR) analysis of mesoderm, endothelial, and hematopoietic marker gene expression throughout the differentiation protocol; (**C**) Schematic of C59 treatment windows and analysis of RUNX1c: Green fluorescent protein (GFP) reporter expression by flow cytometry at d14; (**D**) RUNX1c:GFP^+^ cells normalized to control (0 nM C59, 0.1% dimethyl sulfoxide (DMSO) treated) cells. Tx = treatment; (**E**) %RUNX1c:GFP^+^ (green dot) and %CD34^+^/CD45^+^ (black square) cells in C59 or control (0 nM C59, 0.1% DMSO treated) cells; (**F**) Schematic of CHIR98014 (CHIR) treatment and analysis of RUNX1c:GFP reporter expression by flow cytometry at d14; (**G**) RUNX1c:GFP^+^ cells normalized to control (0 nM CHIR, 0.1% DMSO treated) cells; (**H**) RUNX1c:GFP^+^ cells normalized to control (0 nM CHIR, 0.1% DMSO treated) cells, treated with the indicated dose of CHIR added at days 2–4. Data shown are representative of two biological replicates. Error bars represent standard deviation; * = *p* value < 0.05, ** = *p* value < 0.01, *** = *p* value < 0.001, n.s. = Not significant.

We have previously identified that a β-catenin-mediated Wnt signal is required in zebrafish during a tightly regulated time window as endothelial descendants of posterior lateral mesoderm migrate underneath the somites [36]. The process of deriving hematopoietic progenitors in vitro closely mimics how hematopoietic cells develop in an organism. Pluripotent cells progress through mesodermal and endothelial cell fates before a subset is further specified towards hematopoietic progenitor cells (Figure 1A,B); the corresponding time window for the Wnt requirement in vitro is approximately between day two and day four of differentiation, according to the expression of marker genes for mesoderm (*BRY*, *WNT3*) and endothelium (*CD31/PECAM*) (Figure 1B). We inhibited Wnt signaling using a small-molecule inhibitor of Wnt secretion (C59, [53]) during multiple stages of differentiation (Figure 1C), and analyzed hematopoietic differentiation by flow cytometry for a *RUNX1c:GFP* knock-in reporter which marks hematopoietic stem and progenitor cells [46]. Only Wnt inhibition from days 2–4 significantly decreased differentiation efficiency (Figure 1D). We confirmed this effect on the differentiation outcome by additionally analyzing surface markers CD34 and CD45; the double-labeled population contains hematopoietic progenitors (Figure 1E). These results suggest that similar to the zebrafish and the mouse, Wnt signaling is required during a narrow time window during human in vitro hematopoietic development.

We next sought to determine if ectopic activation of Wnt signaling during this time window could boost hematopoietic differentiation efficiency by treating cells with the Wnt activator CHIR [54,55] during multiple stages of differentiation (Figure 1F). Consistent with Wnt inhibition and previous reports [41,46], we found that only activating Wnt during days 2–4 increased the efficiency of differentiation to RUNX1c:GFP^+^ cells, while Wnt activation during other time windows did not (Figure 1F,G). Consistent with the role of Wnt in stem cell maintenance and differentiation, we also observed a dose-specific effect, where both low and high doses of CHIR were suboptimal for the derivation of hematopoietic progenitors (Figure 1H) [39]. Taken together, these data indicated that Wnt signaling is required in a time- and dose-dependent manner during in vitro development of human pluripotent stem cells to hematopoietic progenitors.

### 3.2. WNT9A Increases the Efficiency of Hematopoietic Progenitor Differentiation in a Time-Dependent Manner

**Figure 2:**
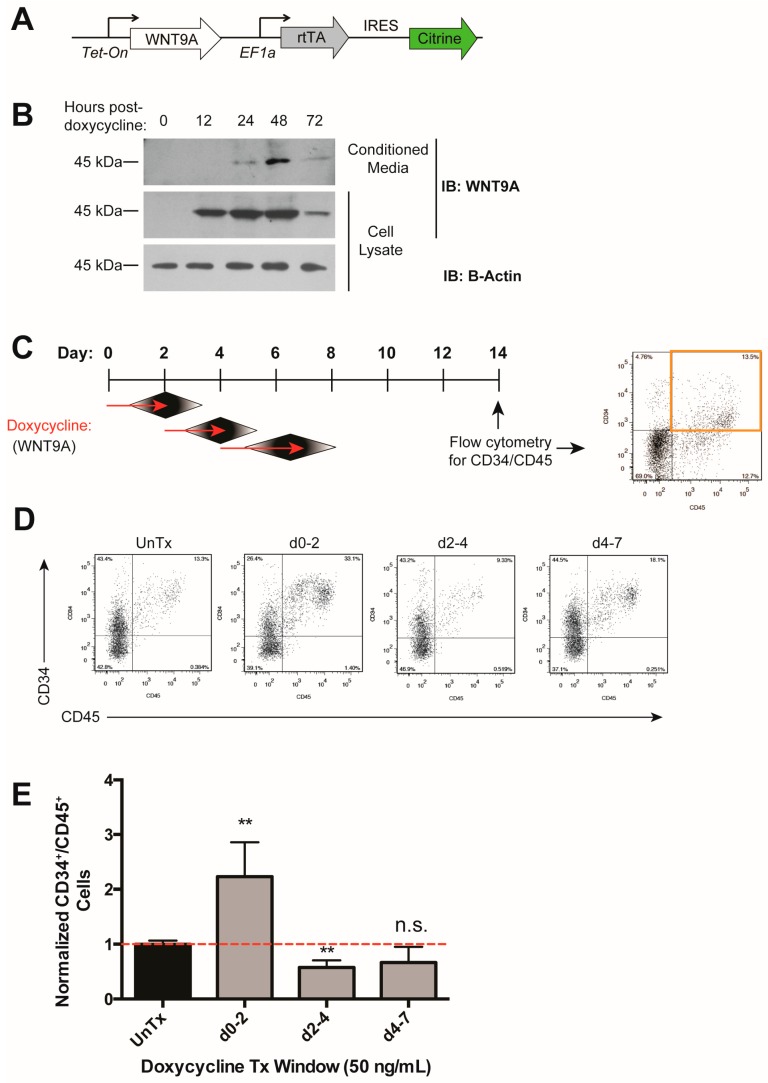
WNT9A increases hematopoietic differentiation efficiency in a time-dependent manner. (**A**) Schematic of inducible WNT9A transgene, which consists of a doxycycline-inducible WNT9A and an internal ribosome entry site (IRES) driving expression of Citrine. (**B**) Immunoblot depicting WNT9A protein expression dynamics in response to 50 ng/mL doxycycline treatment. Cells were treated with doxycycline for 0, 12, 24, or 48 h and analyzed for expression of WNT9A. The 72-h sample was treated with doxycycline for 48 h, then changed to fresh media without doxycycline for 24 h. Top: Conditioned media was pulled down with Blue Sepharose beads and blotted for WNT9A; Middle: Cell lysate was blotted for WNT9A; Bottom: Cell lysate was blotted for β-ACTIN. (**C**) Schematic of doxycycline treatment (Tx) to induce WNT9A expression, and readout of hematopoietic progenitor differentiation by flow cytometry for CD34 and CD45 for the data shown in (**D**,**E**). Red arrows indicate doxycycline treatment, black diamonds indicate the resultant WNT9A protein expression. (**D**) Representative flow cytometry plots for data graphed in (**E**); (**E**) Percentage of CD34^+^/CD45^+^ cells normalized to untreated control. Data represent average of three independent biological replicates; ** = *p* value < 0.01, n.s. = Not significant. Error bars represent standard deviation.

We previously identified Wnt9a to be specifically required during zebrafish HSC development [36]. To determine to what extent the requirement for this Wnt protein is conserved during human hematopoietic development, we generated an hESC cell line (H9) carrying a *WNT9A* transgene under the control of a doxycycline-inducible promoter, and a constitutive promoter driving Citrine expression to monitor silencing of the transgene (Figure 2A). WNT9A secretion peaked at 48 h after doxycycline treatment, and WNT9A persisted in the media for at least 24 h after doxycycline removal (Figure 2B). Upon expression of WNT9A during various time windows of differentiation, we determined that induction of WNT9A from days 0–2 (i.e., WNT9A treatment from days 1–3) resulted in an increase in CD34^+^/CD45^+^ hematopoietic progenitors, while other induction timepoints did not (Figure 2C–E). This is in line with treatment with activation of the Wnt pathway with CHIR from days 2–4, and previous studies indicating that the critical window for Wnt activation during in vitro hematopoietic progenitor differentiation is after the formation of mesoderm from approximately days 2–3 [41,43]. These data demonstrate that WNT9A impacts human hematopoietic progenitor development in a temporally-regulated manner.

### 3.3. WNT9A Stimulates Hematopoietic Differentiation in a Dose-Dependent Manner

**Figure 3:**
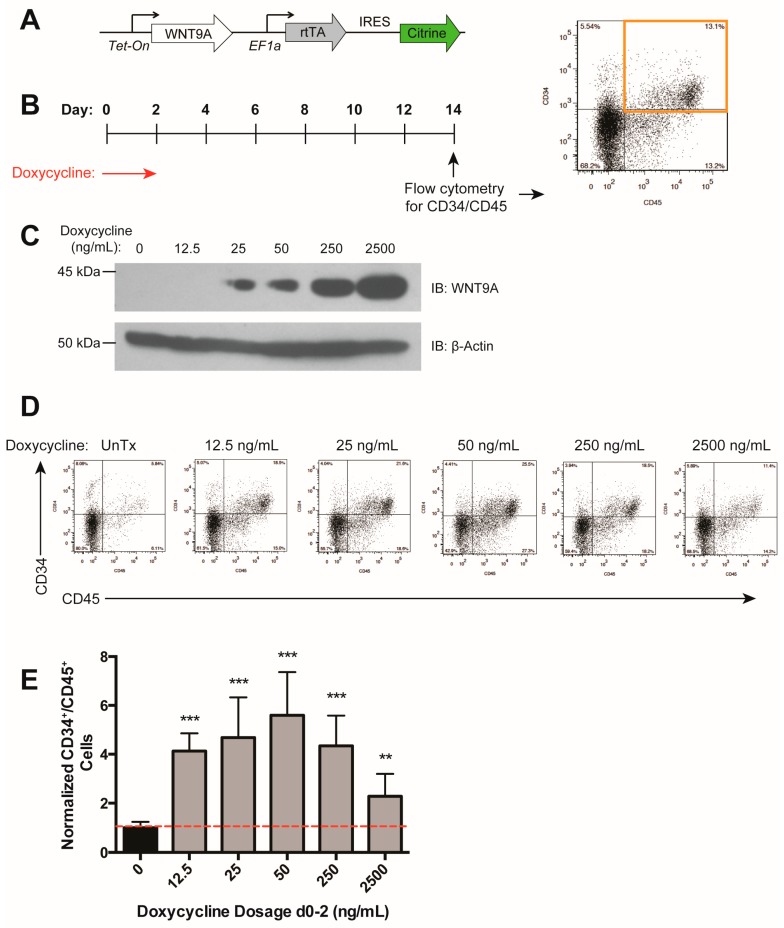
WNT9A has a dose-dependent effect on hematopoietic differentiation efficiency. (**A**) Schematic of doxycycline-inducible *WNT9A* transgene. (**B**) Schematic of doxycycline induction of WNT9A expression and flow cytometry readout for the data shown in (**D**,**E**). (**C**) Immunoblot (IB) showing increasing WNT9A protein expression in response to the dosages of doxycycline shown. Top: WNT9A, Bottom: β-ACTIN loading control. (**D**) Representative flow cytometry plots for data graphed in (**E**); (**E**) Percentage of CD34^+^/CD45^+^ cells normalized to untreated control. Data represent the average of three biological replicates. ** = *p* value < 0.01, *** = *p* value < 0.001, n.s. = Not significant. Error bars represent standard deviation.

The above data indicate that there is an optimal level of Wnt activation that results in maximal increases in differentiation efficiency (Figure 1H). We assessed the dose-dependent effect of WNT9A during days 0–2 of differentiation (Figure 3A,B) and found that while all doses tested resulted in an increase in hematopoietic differentiation efficiency, a maximal effect was reached with a moderate dosage of doxycycline (50 ng/mL), which led to sub-maximal levels of WNT9A protein (Figure 3C–E), consistent with the previously observed ideal level of Wnt signaling (Figure 1G) [39]. Taken together, our data suggest that WNT9A can modulate in vitro human hematopoietic development in a time- and dose-dependent manner.

### 3.4. WNT9A Stimulates Hematopoietic Differentiation in a Paracrine Manner

**Figure 4:**
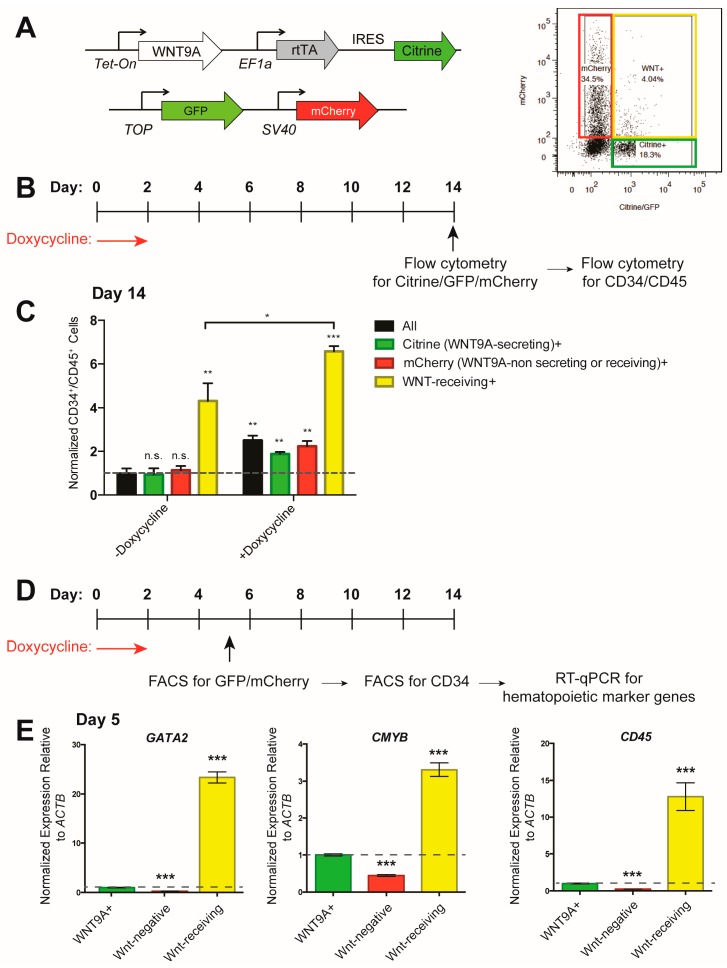
WNT9A signals in a paracrine fashion to instruct differentiation to hematopoietic progenitor cells. (**A**) Schematic of inducible WNT9A and *Tcf optimal promoter (TOP):GFP;SV40:mCherry* transgenes and flow cytometry gating strategy. (**B**) Schematic of doxycycline induction of WNT9A expression, and readout of hematopoietic progenitor differentiation by flow cytometry for GFP/mCherry expression, then subsequent CD34 and CD45 expression for the data shown in (**C**). (**C**) Percentage of CD34^+^/CD45^+^cells in the indicated cell populations normalized to all cells in the untreated control. Data are representative of three biological replicates. (**D**) Schematic of doxycycline induction of WNT9A expression and isolation of CD34^+^ endothelial cells within the indicated cell populations for data shown in (**E**); (**E**) Quantitative reverse transcription polymerase chain reaction (RT-qPCR) for hematopoietic markers *GATA2, CMYB,* and *CD45* in CD34^+^ endothelial cells from the indicated cell populations. Expression values are shown as fold change, normalized to WNT9A-secreting cells (Citrine^+^, green bar). Data represent average of three technical replicates; ** = *p* value < 0.01, *** = *p* value < 0.001, n.s. = Not significant. Error bars represent standard deviation.

In the zebrafish embryo, WNT9A is secreted by the somites and received by the developing hemogenic endothelium in a paracrine manner [36]. To model this, we used the inducible WNT9A hESC line shown in Figure 2 and Figure 3 (constitutively labeled with Citrine), and co-cultured these cells with an hESC line harboring a *TOP:GFP;SV40:mCherry* transgene (constitutively labeled with mCherry) (Figure 4A). This cell line will express GFP after receiving a β-catenin-mediated Wnt cue, and can be distinguished from the WNT9A producing cells by mCherry expression, allowing for analysis of (1) all cells (black bars); (2) WNT9A-secreting cells (Citrine^+^, green bars); (3) non-WNT9A-secreting, non-Wnt-receiving cells (mCherry^+^, red bars); (4) Wnt-receiving cells (GFP^+^/mCherry^+^, yellow bars) (Figure 4A–C). When WNT9A was expressed, differentiation efficiency was increased in all cell populations (Figure 4C). By analyzing hematopoietic markers in sorted CD34^+^ endothelial cells within each of the above populations, we found that Wnt-receiving cells were biased to differentiate to hematopoietic cells as early as day five of the differentiation, since Wnt-receiving endothelial cells are enriched for expression of the hematopoietic marker genes *GATA2*, *CMYB*, and *CD45* (Figure 4D,E). Taken together, these data indicate that during in vitro hematopoietic differentiation, cells must receive a Wnt signal to efficiently differentiate to hematopoietic progenitors. Receiving a WNT9A signal increases this differentiation efficiency. Interestingly, Wnt-receiving cells that received a WNT9A signal differentiated more efficiently than cells that received endogenous Wnt cues. Altogether, this suggests that, like in the zebrafish, there is a requirement for specific Wnt ligands during in vitro differentiation of hematopoietic progenitors.

## 4. Discussion

Wnt signaling has been shown to regulate adult and developmental hematopoiesis in vivo, as well as in the in vitro differentiation of hematopoietic progenitor cells [37]. We recently identified Wnt9a as a specific mediator of a critical Wnt signal required for HSC development in zebrafish [36]. In this study, we sought to extend our findings to human cells and determine to what extent WNT9A can regulate the differentiation of human hematopoietic progenitors from pluripotent precursors. We found that—as in the zebrafish embryo—Wnt/β-catenin signaling positively regulates human hematopoietic differentiation. Similarly, WNT9A improves differentiation efficiency in a time- and dose-dependent manner. We also show that the paracrine nature of the Wnt9a signal identified in the zebrafish occurs in the in vitro hematopoietic differentiation. This is the first report describing a role for WNT9A in mammalian hematopoiesis.

We show that Wnt/β-catenin stimulation improves human hematopoietic progenitor differentiation efficiency, which is consistent with other reports [41,46,56,57,58]. These studies have described an in vitro requirement for Wnt/β-catenin signaling to properly instruct mesodermal fate commitment first to primitive streak-like mesoderm [59], and then further to hematopoietic mesoderm [41,46,58]. This requirement for Wnt/β-catenin signaling is stage-specific; we found that inhibition of Wnt secretion negatively impacted hematopoietic only during certain windows of differentiation. The Wnt pathway is known to have temporally restricted effects on hematopoietic differentiation in vitro; early Wnt stimulation typically stimulates hematopoietic differentiation, and later Wnt stimulation results in differentiation to mesenchymal cells [41,46,60] and cells restricted to intermediate mesoderm [61], which contributes to the urogenital system, and not to the hematopoietic system. The effect of Wnt activation on in vitro hematopoietic progenitor differentiation is dose-dependent, which was previously described in vivo during adult hematopoiesis using an allelic series of APC mutations [39]. Fine-tuning of the level of Wnt activation during in vitro hematopoietic differentiation protocols may be critical for maximizing the number and functionality of hematopoietic progenitors derived from pluripotent precursors.

Herein, we specifically modulated the expression of WNT9A, which resulted in a five-fold increase in CD34^+^/CD45^+^ hematopoietic progenitor cells. In contrast, stimulation of the Wnt pathway using a small-molecule activator CHIR resulted in a two-fold increase in RUNX1c^+^ hematopoietic progenitor cells. In this system, the addition of a specific ligand afforded a larger increase in efficiency than ligand-independent activation of the pathway. This may be partly due to off-target effects that small molecules—such as this potent GSK3 inhibitor—can have on other cellular processes; it is possible that these off-target effects slightly inhibit hematopoietic differentiation. Additionally, GSK3 is a major signaling hub for multiple pathways, including insulin, reelin, neurotransmitter, and Hedgehog signaling; modulation of one of these processes through GSK3 inhibition could potentially decrease hematopoietic differentiation efficiency [62,63,64]. If this is the case, stimulation with a specific Wnt ligand may activate the Wnt/β-catenin signaling cascade more uniquely. The expression of other Wnts, including WNT1, WNT5, WNT3, and WNT3A, were modulated during in vitro hematopoietic differentiation with varying effects on the differentiation outcome, suggesting that specific Wnts may act non-redundantly during differentiation [57,58,60]. This has also been described during in vivo hematopoietic stem cell development [36,40,65]. This may be due to differential Wnt/Fzd binding affinities and the activation of subsequent Fzd-specific gene programs [66,67,68,69]. Interestingly, WNT9A has been described to signal in both a β-catenin-dependent [36,70,71] and -independent [72,73] manner, so it is also possible that WNT9A is additionally activating a β-catenin-independent signaling cascade, which could contribute to the differences in hematopoietic differentiation efficiency between CHIR and WNT9A. Investigation into which ligand(s) most efficiently instruct hematopoietic progenitor fate in vitro may further improve current differentiation protocols.

In zebrafish, Wnt9a is secreted by the somites and received by neighboring cells of the hemogenic endothelium in a paracrine fashion [36]. This signaling mechanism also holds true in vitro; it is the cells that receive a Wnt signal, and not the cells that secrete WNT9A, that differentiate most efficiently to hematopoietic progenitors. This is somewhat surprising, since the spatial architecture that is characteristic of a complex zebrafish embryo does not exist during in vitro hematopoietic differentiation; a small population of WNT9A-secreting cells is not a limiting factor in vitro, as it may be during zebrafish development. Interestingly, Wnt9a has also been described to signal in a paracrine fashion during zebrafish palate morphogenesis and chick hepatic endothelium development [70,74]. This may indicate that the secretion of WNT9A precludes a cell from responding to the Wnt signal in an autocrine manner, and thus largely signals in a paracrine fashion. This differentiation protocol utilizing a co-culture method to study Wnt-secreting and Wnt-receiving cells could prove to be a useful platform in future experiments to dissect the mechanisms of paracrine versus autocrine Wnt signaling, and to better understand the mechanics of Wnt ligand secretion.

Our data indicate that signals identified as regulators of hematopoietic development in zebrafish can be translated to impact human hematopoietic progenitor cells during in vitro models of development. Wnt9a, which is required for HSC development in zebrafish, can be modulated to increase the efficiency of CD34^+^/CD45^+^ hematopoietic cell differentiation from human pluripotent stem cells. These findings represent an important initial step in translating our knowledge of molecular cues governing organismal hematopoiesis to protocols for instructing HSC differentiation in vitro.

## Figures and Tables

**Table 1:** List of quantitative reverse transcription polymerase chain reaction (RT-qPCR) primers used in this study.

Gene Name	Forward (5′→ 3′)	Reverse (5′→ 3′)
*ACTB*	CATCCGTAAAGACCTCTATGCC	ATGGAGCCACCATCCACA
*BRY*	CAGTGGCAGTCTCAGGTTAAGAAGGA	CGCTACTGCAGGTGTGAGCAA
*CD31*	TTCCTGACAGTCTCTTGAGTGG	TTTGGCTAGGCGTGGTTCTCAT
*CD45*	CTCTACGCAAAGCTAGGCCA	ACTTGTCCATTCTGAGCAGG
*CMYB*	GTCACAAATTGACTGTTACAACACCAT	TTCTACTAGATGAGAGGGTGTCTGAGG
*GATA2*	AGCCGGCACCTGTTGTGCAA	TGACTTCTCCTGCATGCACT
*WNT3*	AACAAGCACAACAACGAGGC	CCAGCAGGTCTTCACCTCAC
